# Uncertainty-Aware AI-Assisted Diabetic Retinopathy Grading from Fundus Images with Ordinal Conformal Prediction

**DOI:** 10.3390/diagnostics16162645

**Published:** 2026-08-19

**Authors:** Umar Hasan, Muhammad Ali Nayeem, Turki G. Alghamdi

**Affiliations:** 1 Department of Mathematics and Physics, School of Engineering and Physical Sciences, North South University, Dhaka 1229, Bangladesh; umar.hasan@northsouth.edu; 2 Department of Computer Engineering, College of Computer, Qassim University, Buraydah 51452, Saudi Arabia; 3 Department of Computer Engineering and Networks, College of Computer and Information Sciences, Jouf University, Sakaka 72388, Saudi Arabia; talghamdi@ju.edu.sa

**Keywords:** diabetic retinopathy, fundus photography, artificial intelligence, deep learning, diagnostic support, external validation, uncertainty quantification, conformal prediction, ordinal classification, dataset shift

## Abstract

**Background:** Artificial intelligence (AI) systems for diabetic retinopathy (DR) grading require reliable uncertainty estimates when applied to fundus images outside the development dataset. We evaluated whether conformal prediction can provide structured set-valued outputs and whether internal uncertainty calibration remains reliable during external evaluation. **Methods:** EfficientNet-B0, ResNet-50, and Vision Transformer (ViT-Base) classifiers were trained on APTOS 2019. Split-conformal predictors were calibrated exclusively on held-out APTOS images using three categorical scores, LAC, APS, and RAPS, and an ordinal score restricted to adjacent severity grades. Performance was assessed internally on APTOS and externally on IDRiD, with an independent five-seed ViT replication extending evaluation to Messidor-2, at target coverages of 90% and 95%. **Results:** Coverage was approximately nominal internally but decreased on both external datasets. On IDRiD, the largest deficit occurred for severe DR (grade 3). The ordinal method produced contiguous intervals in 100% of cases and achieved the highest grade-3 coverage in every tested backbone–risk configuration. For ResNet-50 at 95% target coverage, grade-3 coverage increased from 0.750 with APS to 0.945 with the ordinal method, while average set size increased from 2.89 to 3.03. In the independent ViT replication on Messidor-2, ordinal marginal coverage exceeded APS at both targets (0.676 versus 0.632 and 0.741 versus 0.714). **Conclusions:** Internal calibration did not ensure reliable class-specific uncertainty during external evaluation. Ordinal prediction sets improved structural coherence and mitigated severe-grade undercoverage, but did not restore formal coverage guarantees after dataset shift.

## 1. Introduction

Diabetic retinopathy (DR) is assessed from retinal abnormalities visible in fundus photographs and is commonly represented by ordered severity grades ranging from no DR to proliferative disease. Deep learning systems have achieved strong performance for automated DR detection, grading, and retinal referral support [1,2,3,4,5,6,7,8], supporting the broader use of AI-assisted image interpretation in ophthalmology. Recent ophthalmic applications also illustrate how accessible imaging devices and learned visual representations can support disease stratification, including smartphone-assisted corneal imaging for keratoconus assessment [9]. These systems are intended as diagnostic-support tools rather than replacements for clinical assessment, and their outputs must remain interpretable when image quality, acquisition hardware, or patient populations differ from the development setting.

External validation is particularly important in retinal AI because performance can vary across cameras, populations, and screening settings. Cross-camera, multi-disease, African screening, and nationwide Thai evaluations illustrate the range of conditions under which retinal AI must be assessed [10,11,12,13], while transfer from APTOS to IDRiD has produced substantial accuracy degradation in image classifiers [14]. Conventional softmax probabilities may also be poorly calibrated or overconfident under such shifts, motivating Bayesian and sampling-based uncertainty estimation [15,16,17,18]. DR|GRADUATE, for example, jointly estimates disease grade, visual explanation, and predictive uncertainty across several fundus datasets [19]. However, heuristic uncertainty scores do not directly specify how often an uncertainty output should contain the correct grade.

Conformal prediction addresses this limitation by converting classifier probabilities into prediction sets with finite-sample marginal coverage under exchangeability [20,21,22,23]. For a target coverage such as 90%, the output may contain one or several plausible grades rather than a single forced decision. Standard categorical methods, including LAC, APS, and RAPS, nevertheless ignore the ordered structure of DR severity and can return disconnected sets that skip intermediate grades [24,25,26,27]. Moreover, the formal coverage guarantee does not persist automatically when calibration and external evaluation images are not exchangeable. This creates two clinically relevant questions: whether prediction sets remain reliable across datasets, and whether their labels form a coherent interval of adjacent disease severities.

We conducted a retrospective computational study of conformal uncertainty for five-grade DR classification. EfficientNet-B0, ResNet-50, and ViT-Base models were trained on APTOS 2019, conformal predictors were calibrated using held-out APTOS images, and external evaluation was performed on IDRiD and Messidor-2. The primary aim was to determine whether internal marginal coverage translates to external grade-specific reliability; secondary aims were to evaluate prediction-set coherence and size. We evaluated two directional hypotheses: first, that coverage would deteriorate externally and disproportionately affect severe DR because of acquisition and prevalence shifts; and second, that enforcing ordinal intervals would eliminate disconnected outputs and improve severe-grade coverage at the cost of larger sets. The experiments supported these hypotheses while also showing that ordinal structure mitigates, rather than eliminates, external undercoverage.

The key contributions of this study are as follows:

- We evaluate conformal uncertainty for five-grade DR classification by calibrating exclusively on APTOS and testing on two independent external datasets, IDRiD and Messidor-2.
- We show that internally valid marginal coverage can conceal substantial grade-specific undercoverage, particularly for severe DR under changes in image acquisition and disease prevalence.
- We evaluate an ordinal conformal method that represents diagnostic ambiguity as a contiguous interval of adjacent DR grades and compare it with LAC, APS, and RAPS under identical calibration conditions.
- We quantify the trade-off among coverage, severe-grade reliability, prediction-set size, and structural coherence across two convolutional backbones and an independent five-seed ViT replication.

The remainder of the article is organized as follows. Section 2 reviews AI-based ophthalmic image analysis, uncertainty-aware DR grading, and categorical and ordinal conformal prediction. Section 3 describes the datasets, classifier training, conformal calibration, prediction-set methods, study outcomes, and statistical analysis. Section 4 presents the internal and external validation results, including grade-specific undercoverage, set contiguity, and consistency across architectures. Section 5 interprets the findings in relation to prior ophthalmic-AI studies and discusses strengths, limitations, and future research. Section 6 summarizes the principal conclusions.

## 2. Related Work

The study is informed by three related areas: AI-based ophthalmic image analysis, uncertainty-aware DR grading, and conformal prediction for externally evaluated classifiers.

### 2.1. AI-Based Ophthalmic Image Analysis

AI methods are increasingly evaluated as aids for ocular disease detection and stratification from fundus, corneal, and other ophthalmic images. Large retinal studies have demonstrated automated DR detection across diverse populations and clinically oriented referral tasks [1,2,3,4,5], while recent work has explored lower-cost image acquisition for eye-disease assessment [9]. External and real-world studies have evaluated retinal AI across cameras, countries, community screening programs, and mixed retinal diseases [10,11,12,13]. Related medical-imaging studies have likewise emphasized deployment reliability beyond in-distribution performance, including open-set rejection of unseen surgical artifacts and cross-dataset evaluation of parameter-efficient segmentation models [28,29]. These studies show the diagnostic potential of learned image representations but also emphasize the need for validation beyond the development sample. The present study addresses a complementary question: whether the uncertainty attached to an AI-generated DR grade remains reliable when the image dataset and case mix change.

### 2.2. Deep Learning for Diabetic Retinopathy

Deep neural networks have become a common approach for automated DR severity grading. Studies using ResNet, VGG, Inception, transformers, self-supervised learning, and lesion-localization strategies have reported strong performance on fundus-image benchmarks [6,7,8,30,31,32,33,34]. Because DR grades are ordered, methods have also incorporated ordinal or cost-sensitive objectives to penalize clinically larger grading errors. DR|GRADUATE further combined ordinal grading with explanation maps and predictive uncertainty across multiple datasets [19]. Nevertheless, external transfer remains challenging: substantial degradation has been reported between APTOS and IDRiD [14]. Our analysis therefore focuses not on proposing another image backbone, but on evaluating a post hoc uncertainty layer across these datasets.

### 2.3. Conformal Prediction and Distribution Shift

Conformal prediction constructs set-valued outputs with finite-sample marginal coverage under exchangeability [21,22]. Classification methods include the least ambiguous set-valued classifier (LAC) [24], adaptive prediction sets (APS) [25], and regularized APS (RAPS) [26]. In medical imaging, conformal sets have been investigated as a way to communicate predictive uncertainty and support quality-control decisions [35,36]. The guarantee, however, depends on calibration and evaluation observations being exchangeable. Covariate-weighted conformal prediction can address some forms of covariate shift [37], and transductive or graph-based methods have been proposed for medical domain adaptation [38,39,40]. These approaches do not remove the need to report empirical class-specific reliability when both image characteristics and label prevalence change.

### 2.4. Uncertainty Quantification in Retinal Deep Learning

Traditional approaches to uncertainty in diabetic retinopathy grading have heavily explored Bayesian approximations and their variants [41,42,43]. Methods such as Monte Carlo Dropout have been widely adopted to extract predictive confidence alongside severity grading, often integrated with lesion-aware attention networks or generative adversarial augmentations to handle class imbalance [44]. Recent advancements have also integrated Bayesian Convolutional Neural Networks with active learning pipelines to iteratively annotate unlabeled fundus data [45]. Furthermore, ensemble-based Bayesian approaches have been deployed to generate predictive confidence maps, allowing for uncertainty-informed referral strategies that isolate ambiguous cases for expert review [46]. Alternative hybrid methods, such as Deep Kernel Learning, have fused deep feature extractors with Gaussian processes to improve the calibration of out-of-distribution uncertainties and detect overlapping ocular pathologies [47].

Despite demonstrating strong empirical variance estimates and improving standard calibration metrics like the Expected Calibration Error, these heuristic and approximate Bayesian methods do not natively provide distribution-free, finite-sample coverage guarantees. The conformal prediction framework evaluated in our study addresses this specific gap by converting point estimates into set-valued outputs that possess formal marginal coverage guarantees under exchangeability, achieving reliable uncertainty bounds without requiring multiple stochastic forward passes or architectural modifications during inference.

### 2.5. Ordinal Conformal Prediction

Disease-severity labels have a natural order, whereas standard categorical conformal scores can output disjoint sets. Ordinal conformal methods address this mismatch by constructing contiguous intervals and, in some formulations, minimizing interval size [27,48,49]. For DR, an interval such as grades 2–3 communicates uncertainty between adjacent moderate and severe disease, while a disconnected set such as {1,3} is harder to interpret as a diagnostic-support output. We adapt adaptive-mass scoring to preserve this interval structure and evaluate whether it changes severe-grade reliability during external validation.

### 2.6. Summary of Related Work

Table 1 summarizes the clinical–computational gap. Existing DR systems provide accurate point predictions and several forms of heuristic uncertainty, while conformal prediction provides coverage-controlled sets under exchangeability. The unresolved issue is how coherent ordinal sets behave when evaluated on an external fundus dataset with a different grade distribution.

## 3. Materials and Methods

**Figure 1:**
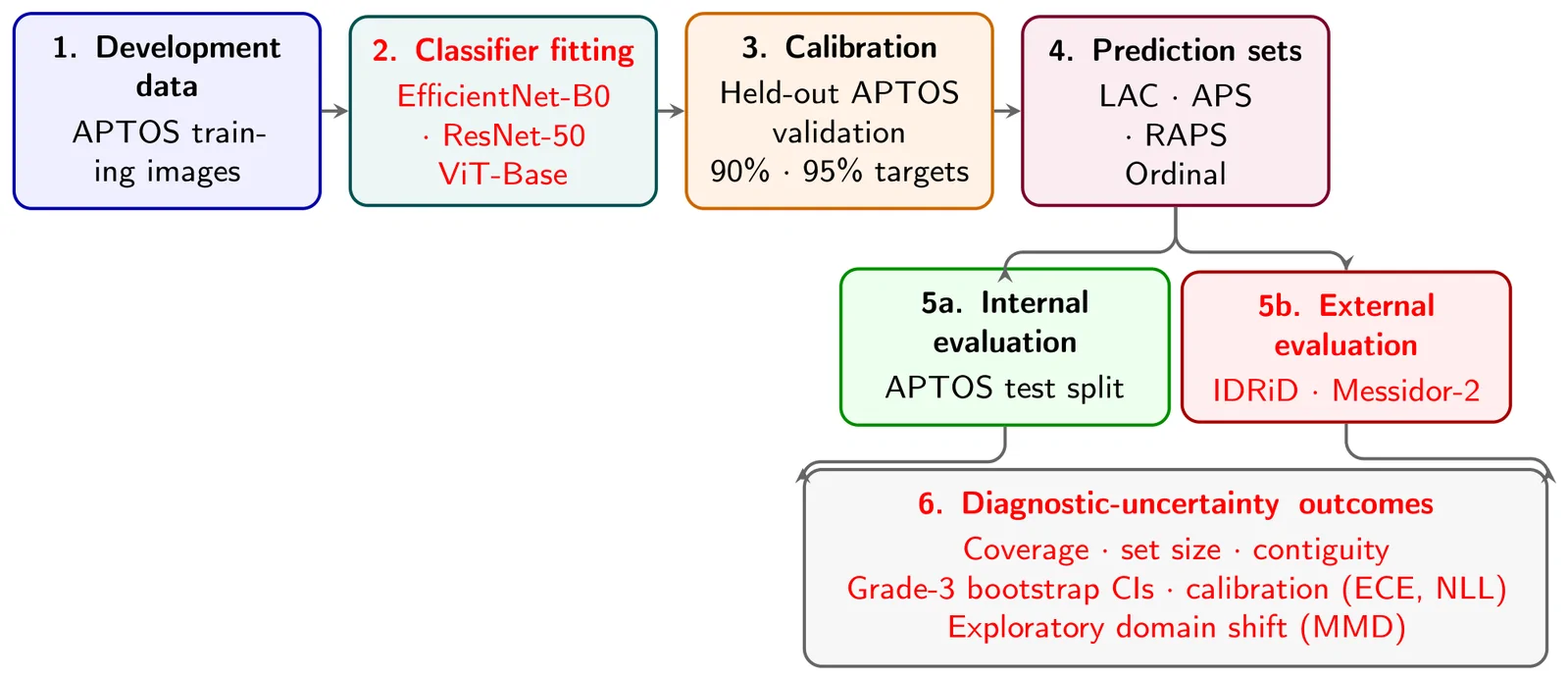
Study workflow. Three classifiers were developed on APTOS training images, prediction-set thresholds were estimated using held-out APTOS validation data, and four uncertainty methods were assessed internally on the APTOS test split and externally on IDRiD and Messidor-2.

This retrospective computational study used publicly available, de-identified fundus-image datasets. As summarized in Figure 1, two convolutional classifiers and a Vision Transformer were trained on APTOS images, uncertainty sets were calibrated on held-out APTOS validation data, and performance was evaluated internally on APTOS and externally on IDRiD and Messidor-2. No external image or label was used for model fitting, early stopping, hyperparameter selection, or conformal calibration.

### 3.1. Study Design and Diagnostic Task

Let X∈X denote a retinal fundus image and Y∈Y={0,1,2,3,4} its DR severity grade, ordered from no DR to proliferative DR. A classifier outputs softmax probabilities $\pi \left(x\right)\in \Delta^{4}$. Instead of reporting only the highest-probability grade, conformal prediction returns a set $C_{\alpha }\left(x\right)\subseteq Y$ of plausible grades at target coverage 1−α. The study examined 90% and 95% target coverage, corresponding to α=0.10 and 0.05.

Because DR severity is ordinal, the structure of the output set is clinically relevant. A set such as {1,2,3} represents uncertainty across adjacent stages, whereas {1,3} skips an intermediate grade. We therefore compared categorical conformal methods with an ordinal method restricted to non-empty intervals {a,a+1,…,b}, where 0≤a≤b≤4. The analysis assessed whether this constraint improved external severe-grade reliability and how much it increased the number of grades returned.

### 3.2. Datasets and External Validation

We study diabetic retinopathy (DR) severity grading, a five-class ordinal task defined on the International Clinical Diabetic Retinopathy Disease Severity Scale (ICDRSS): grade 0 (no DR), grade 1 (mild), grade 2 (moderate), grade 3 (severe), and grade 4 (proliferative). We denote the label space by Y={0,1,2,3,4}, where the natural ordering encodes increasing clinical severity.

**In-distribution data (APTOS 2019).** The APTOS 2019 Blindness Detection dataset comprises 3662 macula-centred fundus photographs acquired by Aravind Eye Hospital (India) under heterogeneous conditions and graded by clinicians on the ICDRSS. We use the publicly available train/validation/test partition, yielding 2930 training, 366 validation, and 366 test images. The class distribution is markedly imbalanced: in the training split, the five grades contain 1434, 300, 808, 154, and 234 images, respectively, so that severe DR (grade 3) is the rarest class (≈5%). We use the training split to fit the classifier, the validation split as the conformal *calibration* set, and the test split for in-distribution evaluation.

**Out-of-distribution data (IDRiD).** The Indian Diabetic Retinopathy Image Dataset (IDRiD) [50] provides fundus images acquired at an Indian eye clinic with a $50^{\circ }$ field-of-view camera at 4288×2848 resolution. From the disease-grading subset, we use all 455 graded images, with grade counts 129, 22, 156, 84, and 64; severe DR (grade 3) accounts for 84 images (≈18%). IDRiD is used exclusively for out-of-distribution (OOD) inference: no IDRiD image is seen during training or calibration.

**Nature of the shift.** APTOS and IDRiD differ both in acquisition (camera, field of view, population) and, critically, in *label prior*: the in-distribution data are dominated by grade 0, whereas IDRiD is enriched for moderate-to-severe disease. This combined covariate-and-label shift is central to our analysis, since the standard conformal coverage guarantee (Section 3.4) requires exchangeability between calibration and test data, which the shift violates.

**Additional Out-of-Distribution Data (Messidor-2).** To determine whether the external findings extended beyond IDRiD, we used Messidor-2 as a secondary evaluation cohort. The public image collection was paired with the distributed adjudicated DR labels used in prior retinal-AI evaluation [1]. After retaining images marked as adjudicated gradable, the cohort contained 1744 images with grade counts 1017, 270, 347, 75, and 35. Thus, Messidor-2 provides a geographically distinct external cohort, although its severe-grade sample (n=75) is not larger than that of IDRiD (n=84). The Messidor-2 experiment was conducted as an independent five-seed ViT replication using the same APTOS-only development protocol.

### 3.3. AI Classifiers and Training

To assess whether our conclusions are architecture-specific, we trained three distinct backbones initialized from ImageNet-pretrained weights: a lightweight EfficientNet-B0, a ResNet-50, and a Vision Transformer (ViT-Base). Each backbone terminates in a five-way linear classification head, and softmax outputs $\pi \left(x\right)\in \Delta^{4}$ are used as the base scores for conformal calibration.

Standardized preprocessing was applied to all images. Images were converted to RGB, resized to 384×384 pixels, converted to tensors, and normalized using standard ImageNet channel statistics. Training augmentation comprised horizontal and vertical flips, random rotation ($\pm 15^{\circ }$), and color jitter; evaluation used deterministic resizing and normalization.

Networks were optimized with AdamW under a cosine-annealed schedule for up to 15 epochs. Because the APTOS dataset is highly imbalanced (e.g., severe DR represents only 5% of the training split), we explicitly applied class-weighted cross-entropy loss during training. This technique penalizes errors on minority classes heavily, ensuring the classifier learns features for rare, severe disease stages rather than defaulting to the majority “No DR” class. A 10% stratified hold-out carved from the training split was used for early stopping (patience 4).

The class weight for grade *c* was $w_{c}=N/\left(Kn_{c}\right)$, where *N* is the training split size, K=5, and $n_{c}$ is the number of training images in grade *c*. Training used a batch size of 8, an initial learning rate of $3\times 10^{-4}$, weight decay of $10^{-4}$, and mixed-precision computation. The archived experiments used PyTorch 2.10.0+cu128 and timm 1.0.26 in a CUDA-enabled Kaggle runtime. The accelerator identifier and peak allocated memory were not retained in the notebook metadata; we therefore report measured inference times rather than an unverifiable hardware specification.

### 3.4. Split Conformal Prediction

Split (inductive) conformal prediction [21,22] converts any trained classifier into a set-valued predictor with a finite-sample coverage guarantee. Let s:X×Y→R be a *nonconformity score*, where larger s(x,y) indicates that label *y* conforms less well to input *x*. Given a calibration set ${\left\{\left(X_{i},Y_{i}\right)\right\}}_{i=1}^{n}$ and a target miscoverage level α∈(0,1), define the conformal threshold

(1) $$\hat{q}\;=\;\mathrm{the}\;\frac{\left\lceil (n+1)(1-\alpha )\right\rceil }{n}\;\mathrm{empirical}\;\mathrm{quantile}\;\mathrm{of}\;{\left\{s\left(X_{i},Y_{i}\right)\right\}}_{i=1}^{n},$$

with $\hat{q}=+\infty$ when ⌈(n+1)(1−α)⌉>n. The prediction set for a test input *x* is

(2) $$C_{\alpha }\left(x\right)\;=\;\left\{\;y\in Y:s\left(x,y\right)\leq \hat{q}\;\right\}.\;$$

If the calibration observations and a future test observation are exchangeable, the standard split-conformal argument gives

(3) $$P\;\left(Y_{n+1}\in C_{\alpha }\left(X_{n+1}\right)\right)\geq 1-\alpha \;$$

for any fixed nonconformity score [21,22,25]. This is a population-level marginal statement; it does not ensure the same coverage within every DR grade. It also does not apply automatically to IDRiD because the external dataset differs from APTOS in image acquisition and label prevalence. We therefore report empirical marginal and grade-conditional coverage separately.

### 3.5. Theoretical Guarantee of the Ordinal Score

A primary methodological requirement of any conformal score is that it preserves the finite-sample marginal coverage guarantee under exchangeability. We formalize this for the proposed ordinal method.

**Proposition** **1.** *Condition on the fitted classifier and let ${\left\{\left(X_{i},Y_{i},U_{i}\right)\right\}}_{i=1}^{n+1}$ be exchangeable, where the tie-breaking variables $U_{i}\overset{\mathrm{iid}}{∼}\mathrm{Unif}\left(0,1\right)$ are independent of the observations. If $C_{\alpha }\left(x\right)$ is obtained by inverting the ordinal nonconformity score in Algorithm 1 at the threshold $\hat{q}$ in Equation (1), then*

$$P\;\left\{Y_{n+1}\in C_{\alpha }\left(X_{n+1}\right)\right\}\geq 1-\alpha .$$

**Proof.**  For fixed classifier probabilities, the greedy expansion from the modal grade defines an ordering of the labels that depends only on *x*. For any candidate label *y*, the ordinal score is the probability mass of labels preceding *y* in this ordering plus the randomized contribution $\left(1-U\right)\pi_{y}\left(x\right)$. It is therefore a measurable scalar score s(x,y,u). The augmented calibration scores $S_{i}=s\left(X_{i},Y_{i},U_{i}\right)$ and test score $S_{n+1}=s\left(X_{n+1},Y_{n+1},U_{n+1}\right)$ are exchangeable. Hence, the rank of $S_{n+1}$ among $S_{1},\ldots ,S_{n+1}$ is uniform after random tie-breaking, and the split-conformal quantile in Equation (1) gives $P(S_{n+1}\leq \hat{q})\geq 1-\alpha$. Algorithm 1 includes exactly the prefix of the same expansion ordering whose randomized scores do not exceed $\hat{q}$; therefore, $S_{n+1}\leq \hat{q}$ is equivalent to $Y_{n+1}\in C_{\alpha }\left(X_{n+1}\right)$. The interval restriction changes the score ordering but not the exchangeability argument.    □

### 3.6. Nonconformity Scores

All four scores below use the same calibration procedure in Equations (1) and (2). Under exchangeability they share the marginal coverage property in Equation (3); they differ in the shape and size of their outputs. For a fixed input *x*, let $\pi_{\left(1\right)}\geq \pi_{\left(2\right)}\geq \cdots \geq \pi_{\left(K\right)}$ denote the softmax probabilities sorted in descending order, with K=|Y|=5.

**LAC.** The least-ambiguous set-valued classifier [24] uses

(4) $$s_{\mathrm{LAC}}\left(x,y\right)=1-\pi_{y}\left(x\right),\;$$

producing the smallest sets but poor conditional coverage.

**APS.** Adaptive prediction sets [25] accumulate sorted probability mass. If label *y* has rank *r* in the descending order, then, with u∼Unif(0,1),

(5) $$s_{\mathrm{APS}}\left(x,y\right)=\sum_{j<r}\pi_{\left(j\right)}\left(x\right)+u\;\pi_{\left(r\right)}\left(x\right).$$

**RAPS.** Regularized APS [26] adds a size penalty that discourages large sets,

(6) $$s_{\mathrm{RAPS}}\left(x,y\right)=\sum_{j<r}\pi_{\left(j\right)}\left(x\right)+u\;\pi_{\left(r\right)}\left(x\right)+\lambda \;{\left(r-k_{\mathrm{reg}}\right)}^{+},$$

with hyperparameters $k_{\mathrm{reg}}\in N$ and λ≥0 selected as described in Section 3.7.

**Ordinal contiguous score.** The three scores above may return non-contiguous sets such as {2,4} on an ordered severity scale. We adapt the adaptive-mass construction to grow a contiguous interval of grades. Starting from the modal grade $m=\arg\max_{y}\pi_{y}\left(x\right)$, the interval expands outward by absorbing the higher-probability adjacent grade until the target label is included. The score is the probability mass accumulated at that point, with uniform randomization on the last included grade. Algorithm 1 describes calibration and prediction. Because each expansion adds only a neighboring grade, every output is a contiguous interval, while the standard split-conformal coverage continues to apply under exchangeability.
**Algorithm** **1** Ordinal contiguous nonconformity score and prediction set**Require:** softmax vector $\pi \in \Delta^{K-1}$; (*y* for calibration) or ($\hat{q}$ for prediction); u∼Unif(0,1)
  1: $m\leftarrow \arg\max_{k}\pi_{k}$;   [a,b]←[m,m];   M←0
 **(a)** **Calibration score** for true label *y*:
  2: **if** y=m **then** **return** $M+\left(1-u\right)\;\pi_{m}$  3: **end if**  4: $M\leftarrow \pi_{m}$  5: **while** y∉[a,b] **do**  6:       j← neighbour of [a,b] (i.e., a−1 or b+1) with larger $\pi_{j}$  7:       **if** j=y **then return** $M+\left(1-u\right)\;\pi_{y}$  8:       **end if**  9:       $M\leftarrow M+\pi_{j}$;   extend [a,b] to include *j*10: **end while**
 **(b)** **Prediction set** at threshold $\hat{q}$:
11: include *m* if $\left(1-u\right)\pi_{m}\leq \hat{q}$; then repeatedly absorb the larger-probability neighbour *j* while $M+\left(1-u\right)\pi_{j}\leq \hat{q}$, updating $M\leftarrow M+\pi_{j}$ and [a,b], and stop at the first violation.12:  **return** the interval {a,…,b}


For APS, RAPS, and the ordinal score, we adopt the standard randomised formulation for exact coverage and, following common practice, force each set to be non-empty by retaining the top-ranked grade when the threshold would otherwise exclude all labels.

**Computational complexity.** For *K* grades, LAC requires O(K) comparisons. APS and RAPS require sorting the *K* probabilities and therefore cost O(KlogK) per image. The ordinal method identifies the modal grade and visits each adjacent grade at most once, giving O(K) time and O(K) output storage. With K=5, all four post hoc procedures are negligible relative to neural-network inference.

### 3.7. Baseline Hyperparameter Selection

To avoid handicapping the RAPS baseline with arbitrary hyperparameters, we tune $\left(k_{\mathrm{reg}},\lambda \right)$ per backbone, per α, and per seed by minimizing average set size. The calibration set is partitioned 70/30 into a fitting and a tuning subset (stratified by grade); for each candidate on the grid $k_{\mathrm{reg}}\in \left\{1,2,3\right\}$ and λ∈{0,0.001,0.01,0.05,0.1,0.2,0.5,1.0}, the threshold is computed on the fitting subset and the resulting average set size measured on the tuning subset, retaining the smallest-size configuration whose tuning coverage is at least 1−α−0.03. The selection uses an independent random stream so that the LAC, APS, and ordinal results are unaffected; the chosen values vary across seeds and no single λ dominates. We explicitly confirm that all hyperparameters, including the RAPS regularization penalties and early-stopping criteria, were fixed exclusively using the internal APTOS validation split. No images or labels from the external IDRiD or Messidor-2 datasets were observed by the model or the conformal calibration routines at any point prior to final evaluation, ensuring strict isolation between development and external testing.

### 3.8. Study Outcomes and Metrics

The primary outcome was empirical marginal coverage. Secondary outcomes were average set size, grade-conditional coverage, and the contiguity fraction. Severe-grade (grade 3) coverage was prespecified as a focus because of its clinical importance and prevalence shift.

To complement conformal coverage, we evaluated Expected Calibration Error (ECE; 15 equal-width confidence bins) and Negative Log-Likelihood (NLL) from the underlying softmax probabilities. These classifier-level metrics are identical across conformal scores because the post hoc methods do not alter the base probabilities. As an exploratory description of covariate shift, we computed Maximum Mean Discrepancy (MMD) with the default radial-basis-function kernel on fixed ImageNet-pretrained ViT features and visualized those features using t-SNE (perplexity 30, random seed 42). Finally, we measured the wall-clock overhead of prediction-set generation.

### 3.9. Statistical Analysis

Class-conditional coverage on rare grades is estimated from small denominators (e.g., 84 IDRiD grade-3 images). To make this sampling uncertainty explicit, we compute 95% confidence intervals by a two-way cluster bootstrap that resamples, with replacement, both evaluation images and training seeds (B=5000 resamples, 2.5/97.5 percentiles), thereby propagating both finite-sample and model-training variability. For the independent ViT replication, the prespecified directional hypothesis that ordinal coverage exceeds each categorical baseline was assessed using an exact one-sided Wilcoxon signed-rank test on the five paired seed-level grade-3 coverage estimates. Given the small number of seeds, these *p*-values are interpreted as supportive rather than definitive evidence.

## 4. Results

Throughout, target coverage is 1−α; “ID” denotes internal evaluation on the APTOS test split and “OOD” denotes external evaluation on IDRiD, consistent with the labels embedded in the figures. All values are means over five seeds ± one standard deviation.

### 4.1. Internal Validation on APTOS

**Figure 2:**
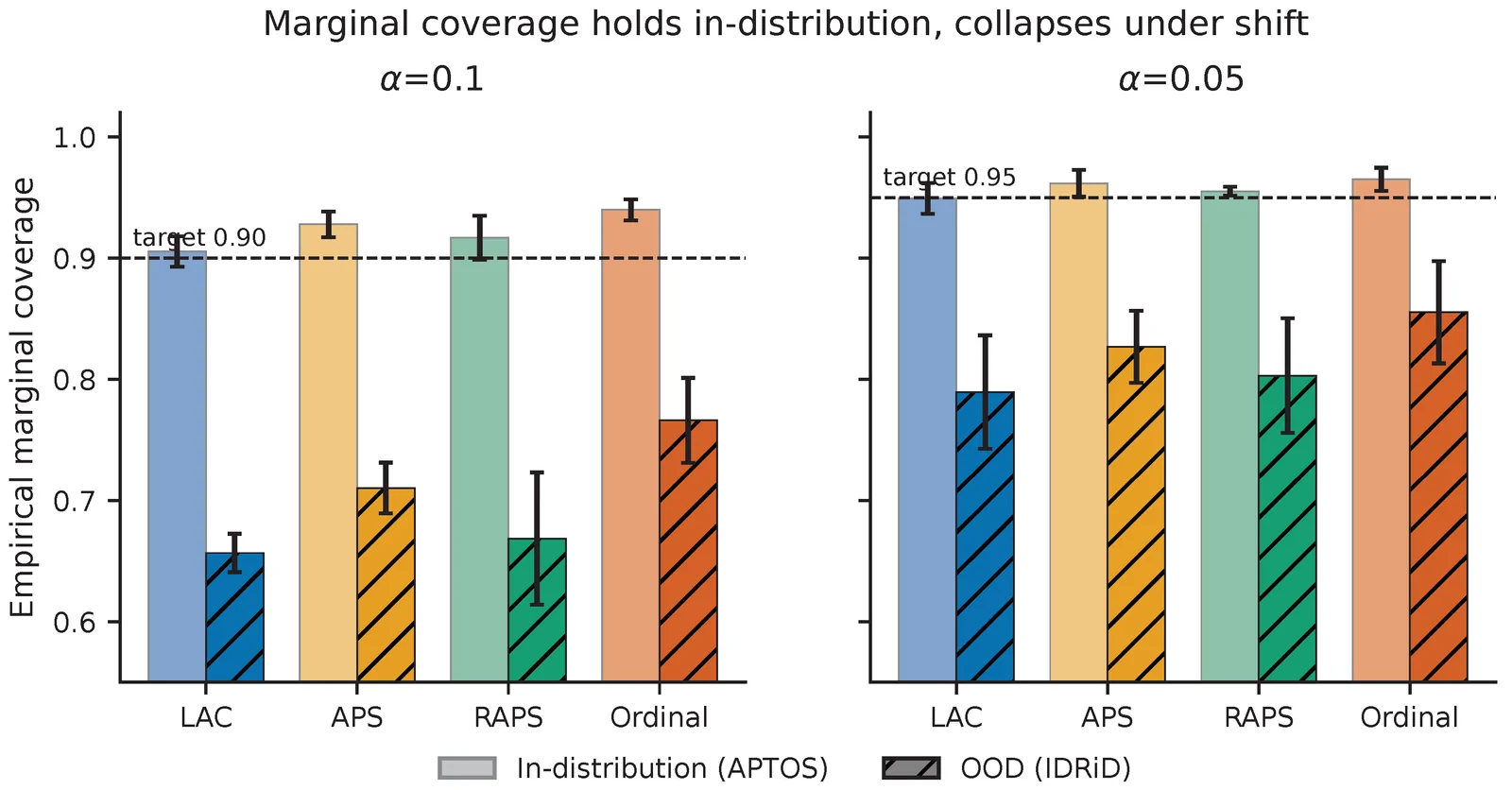
Empirical marginal coverage on the APTOS test split (ID) and IDRiD (OOD) for each score, at α=0.10 (**left**) and α=0.05 (**right**). Bars show means over five seeds, with numerical values displayed directly above the bars.

On the APTOS test split, every method attained its target coverage for both backbones and both risk levels (Table 2, Figure 2). For EfficientNet-B0 at α=0.10, marginal coverage ranged from 0.905±0.013 (LAC) to 0.940±0.009 (Ordinal) against the 0.90 target; for ResNet-50 the range was 0.917±0.011 to 0.958±0.021. LAC was closest to the nominal level, whereas the adaptive methods were mildly conservative, partly because all outputs were constrained to be non-empty. The internal results confirm that the uncertainty calibration achieved its intended population-level coverage on held-out images from the development dataset.

### 4.2. External Validation on IDRiD

When the APTOS-calibrated predictors were evaluated on IDRiD, marginal coverage fell below target in every configuration (Table 2, Figure 2). The deficit was greatest for EfficientNet-B0: at α=0.10, LAC coverage decreased to 0.657±0.016, a 24-percentage-point shortfall, while APS, RAPS, and Ordinal achieved 0.710, 0.669, and 0.766, respectively. ResNet-50 retained higher external coverage; its best categorical method (APS) reached 0.907±0.050 at α=0.05. Thus, uncertainty degradation occurred for both classifiers, but its magnitude depended on the underlying image model.

### 4.3. Quantitative Analysis of Domain Shift

**Figure 3:**
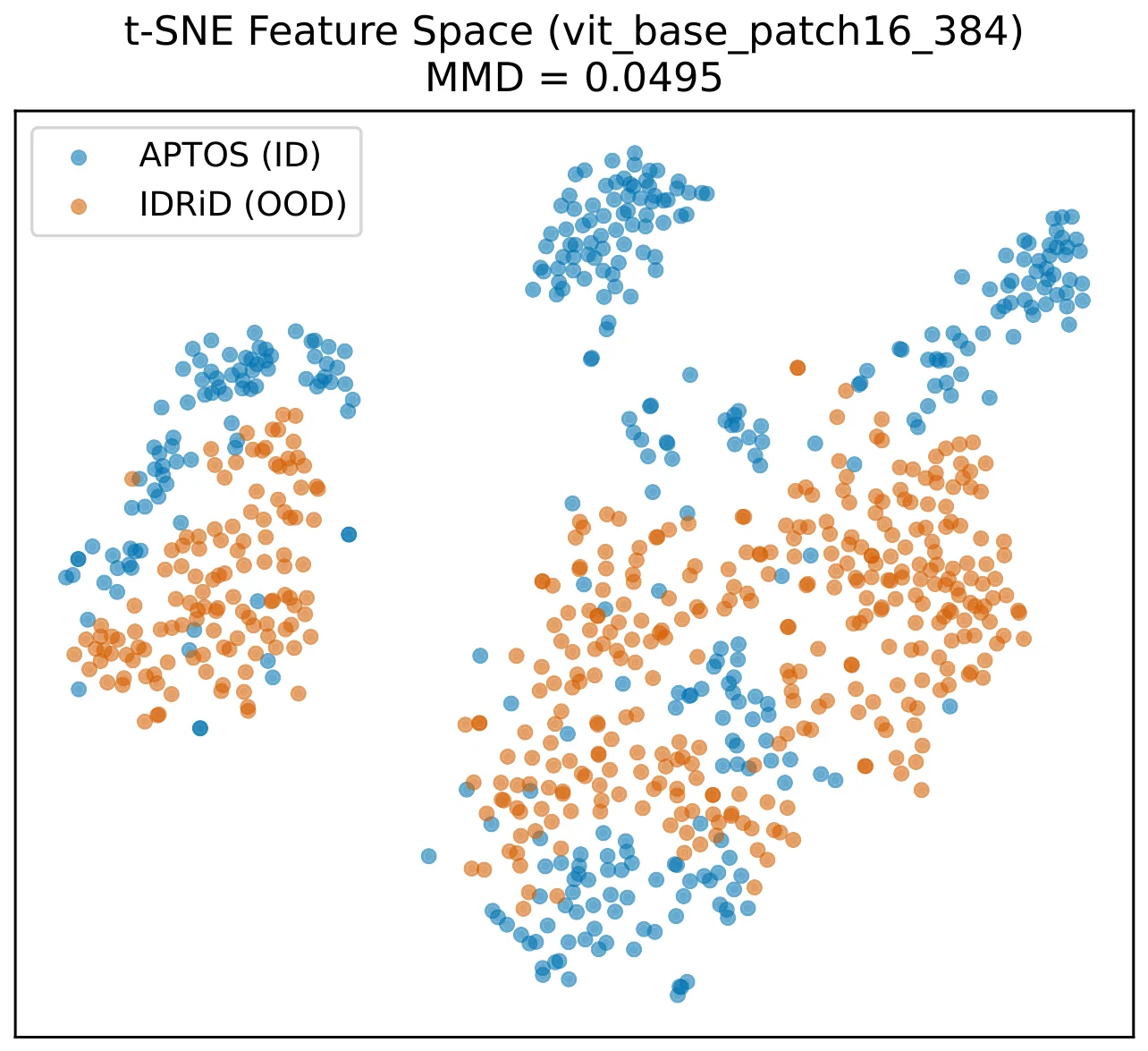
Exploratory t-SNE visualization of fixed ImageNet-pretrained ViT-Base features for the APTOS test split and IDRiD. The corresponding descriptive MMD value was 0.0495; neither t-SNE nor the uncalibrated MMD value is interpreted as a formal causal test of the coverage degradation.

An exploratory representation-space analysis provided additional evidence that APTOS and IDRiD differ in image characteristics. Fixed ImageNet-pretrained ViT features yielded an MMD value of 0.0495, and the two-dimensional t-SNE projection (Figure 3) showed partial domain separation. Because the feature extractor was not fine-tuned for DR and no inferential calibration was attached to the raw MMD value, these analyses are descriptive rather than a formal test of whether dataset shift caused the observed coverage loss.

### 4.4. Classifier Calibration Under External Evaluation

Calibration metrics from the independent five-seed ViT replication are summarized in Table 3. NLL increased markedly from APTOS to both external datasets, whereas ECE did not change monotonically, illustrating that a single calibration summary may not track external coverage failure. These quantities describe the shared base classifier and therefore do not differ across LAC, APS, RAPS, and the ordinal post hoc score.

### 4.5. Statistical Significance of Severe-Grade Reliability

In the independent ViT replication, grade-3 coverage was higher for the ordinal method than for every categorical baseline in all five paired seeds. Against APS, the mean difference was +0.357 at α=0.10 (exact one-sided Wilcoxon p=0.0312) and +0.160 at α=0.05 (p=0.0625). Thus, the directional test supports the improvement at the 90% target and provides weaker evidence at the 95% target. The small number of seeds and one-sided alternative should be considered when interpreting these results.

### 4.6. Computational Overhead

ViT-Base forward-pass inference required approximately 59–73 ms per image across APTOS and IDRiD in the archived Kaggle runs. Prediction-set generation required approximately 0.001 ms per image for APS and RAPS and 0.007–0.011 ms for the ordinal method. These timings indicate negligible post hoc overhead relative to neural-network inference, but they should not be interpreted as a hardware-independent deployment benchmark.

### 4.7. External Undercoverage Concentrates in Severe DR

**Figure 4:**
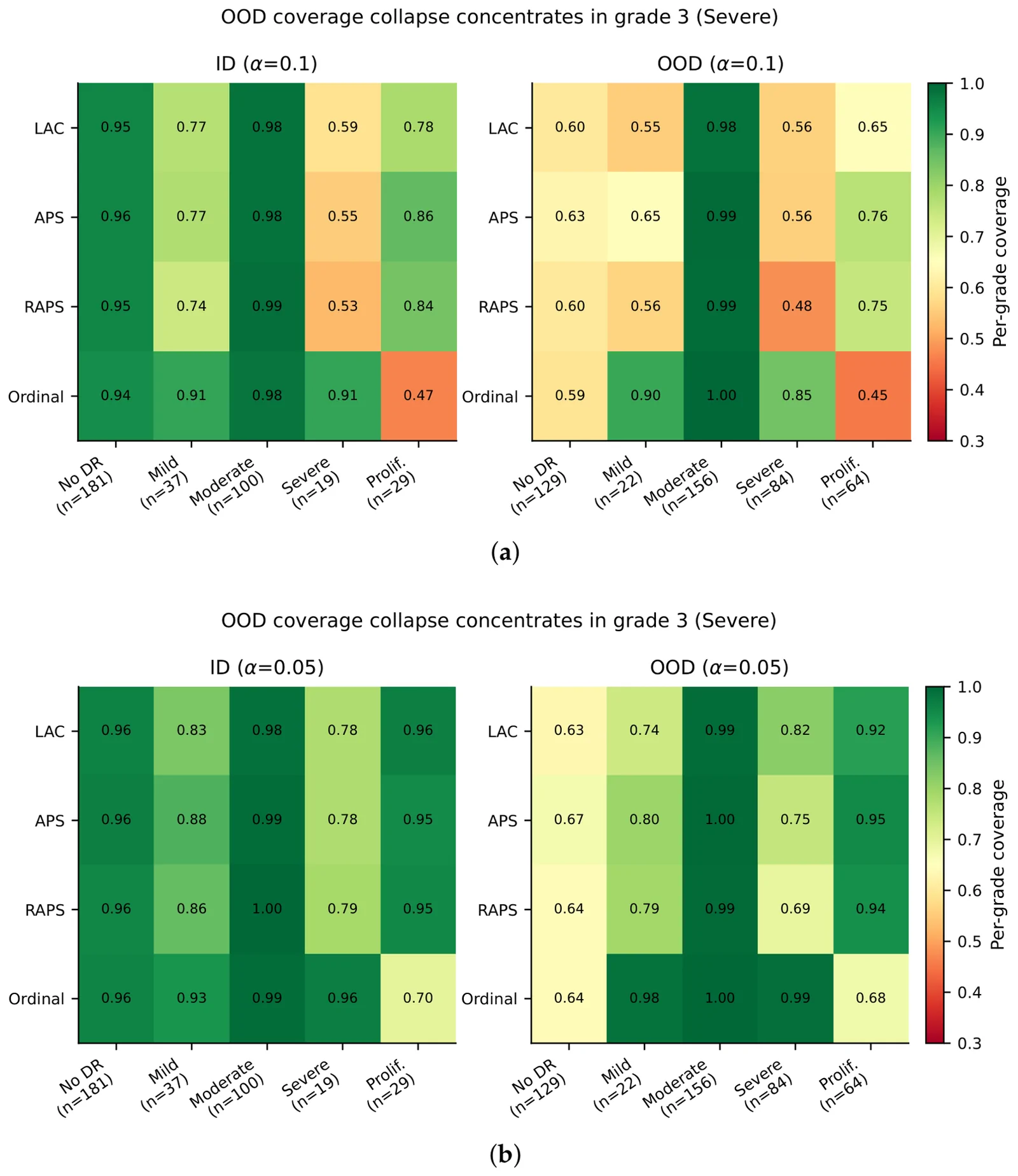
Class-conditional coverage per grade (rows: methods; columns: grades) for internal and IDRiD evaluation. (**a**) α=0.10; (**b**) α=0.05. The x-axis labels report the number of images in each grade; the external coverage deficit concentrates strongly in grade 3 for the non-ordinal scores.

Grade-conditional analysis localized the largest external failure to grade 3 (severe DR; Figure 4). For EfficientNet-B0 at α=0.10, grade-3 coverage was 0.302 with LAC, 0.431 with APS, and 0.333 with RAPS, substantially below coverage for the more common grades 0–2. ResNet-50 showed the same pattern (0.452, 0.636, and 0.526, respectively). Grade 3 represented approximately 5% of the APTOS training set but 18% of IDRiD. Marginal coverage concealed this class-specific weakness: with ResNet-50 at α=0.05, APS achieved external marginal coverage of 0.907 while grade-3 coverage was only 0.750.

### 4.8. Ordinal Sets Improve Structural Coherence

**Figure 5:**
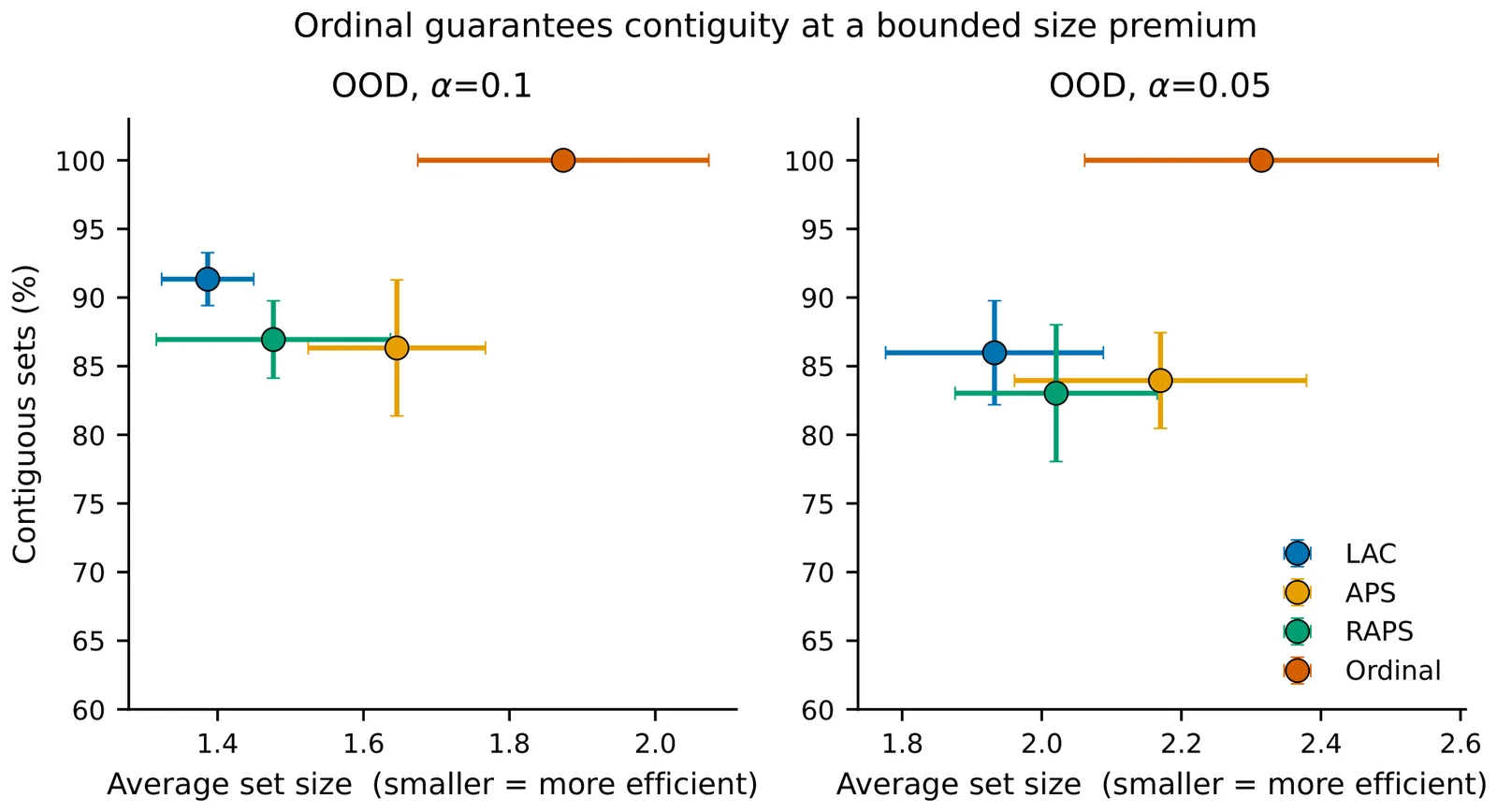
Efficiency–interpretability trade-off on IDRiD (OOD): average set size versus contiguity fraction. The ordinal score attains 100% contiguity at a modest size premium over the adaptive baselines.

The ordinal score produces contiguous severity intervals in 100% of cases across all conditions (contiguity =1.000±0.000), whereas the baselines emit non-contiguous sets at appreciable rates; for EfficientNet-B0 at α=0.05, contiguity is 0.895 (APS), 0.903 (LAC), and 0.884 (RAPS), and RAPS on ResNet-50 falls to 0.796 at α=0.05. This guarantee is not free: the ordinal score yields the largest average sets in every configuration, for EfficientNet-B0 OOD at α=0.10, 1.87 grades versus 1.65 (APS) and 1.39 (LAC), so its benefit is a Pareto trade-off of guaranteed interpretability against a modest size premium (Figure 5), not strict dominance.

### 4.9. Ordinal Sets Improve Severe-Grade Reliability

**Figure 6:**
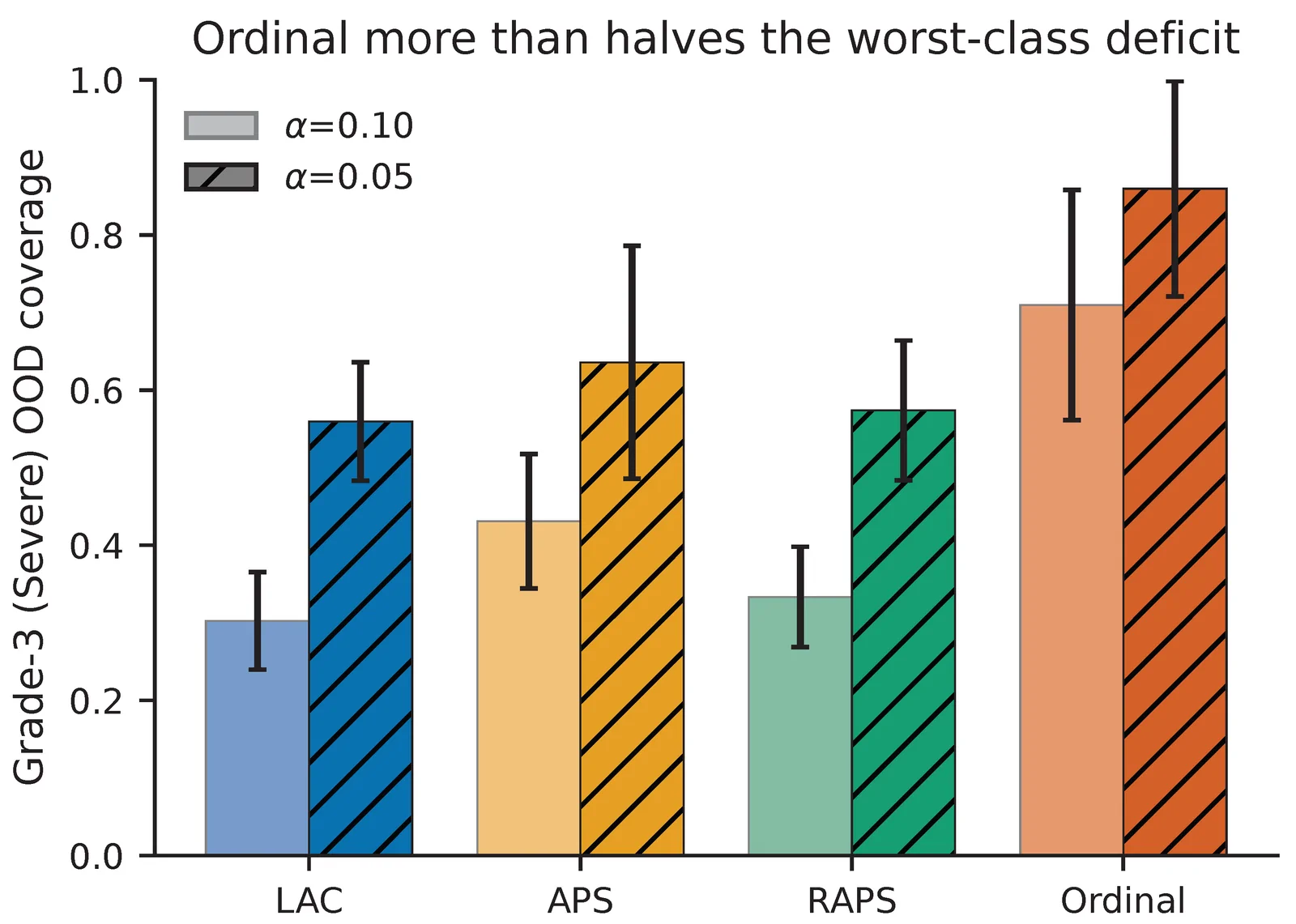
Grade-3 (severe DR) IDRiD coverage by method at both risk levels for EfficientNet-B0. Bars show means over five seeds, with numerical values displayed directly above the bars.

**Figure 7:**
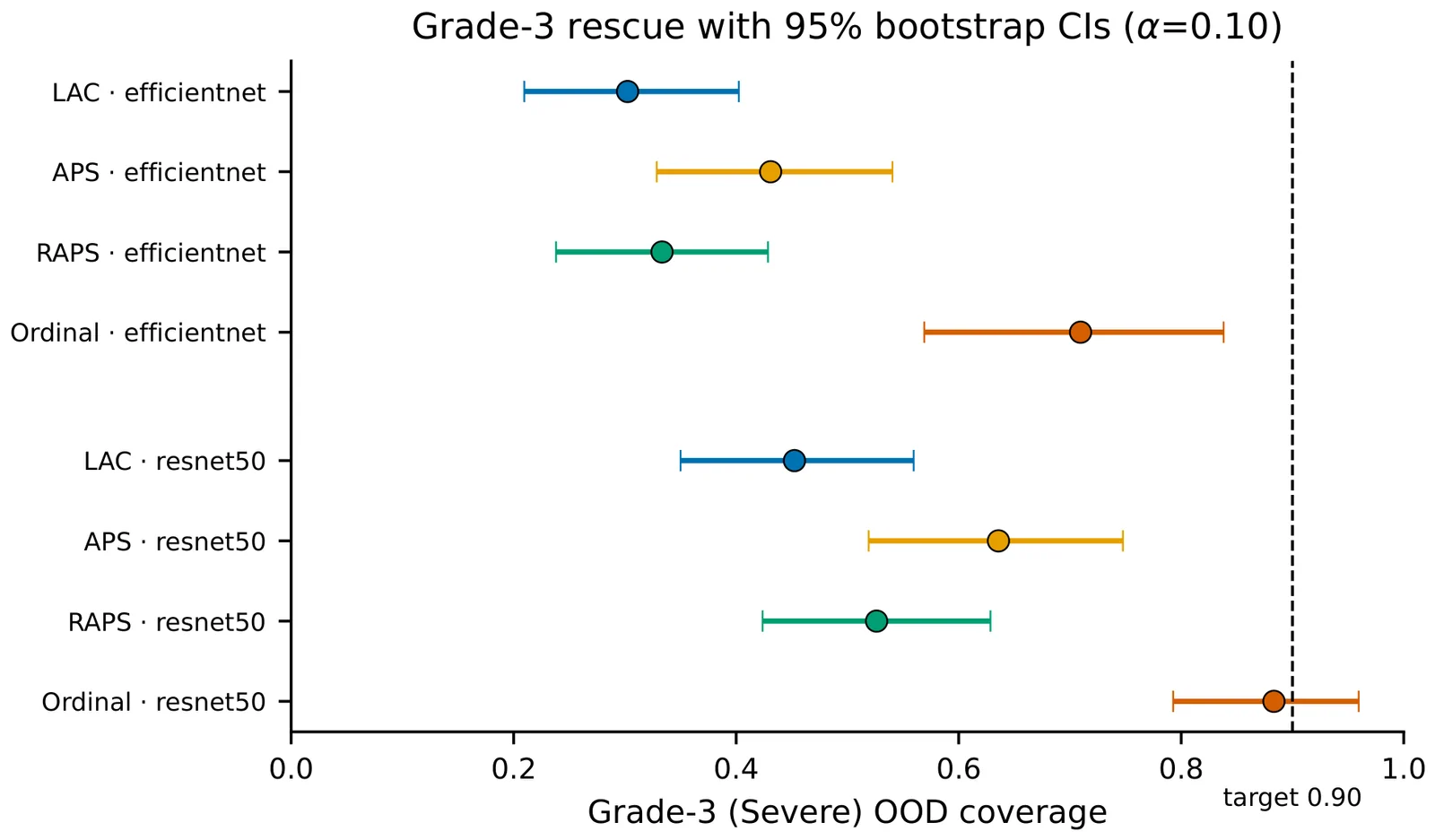
Grade-3 OOD coverage with 95% two-way cluster bootstrap confidence intervals (α=0.10) across methods and backbones. The ordinal interval is separated from the baselines’.

**Figure 8:**
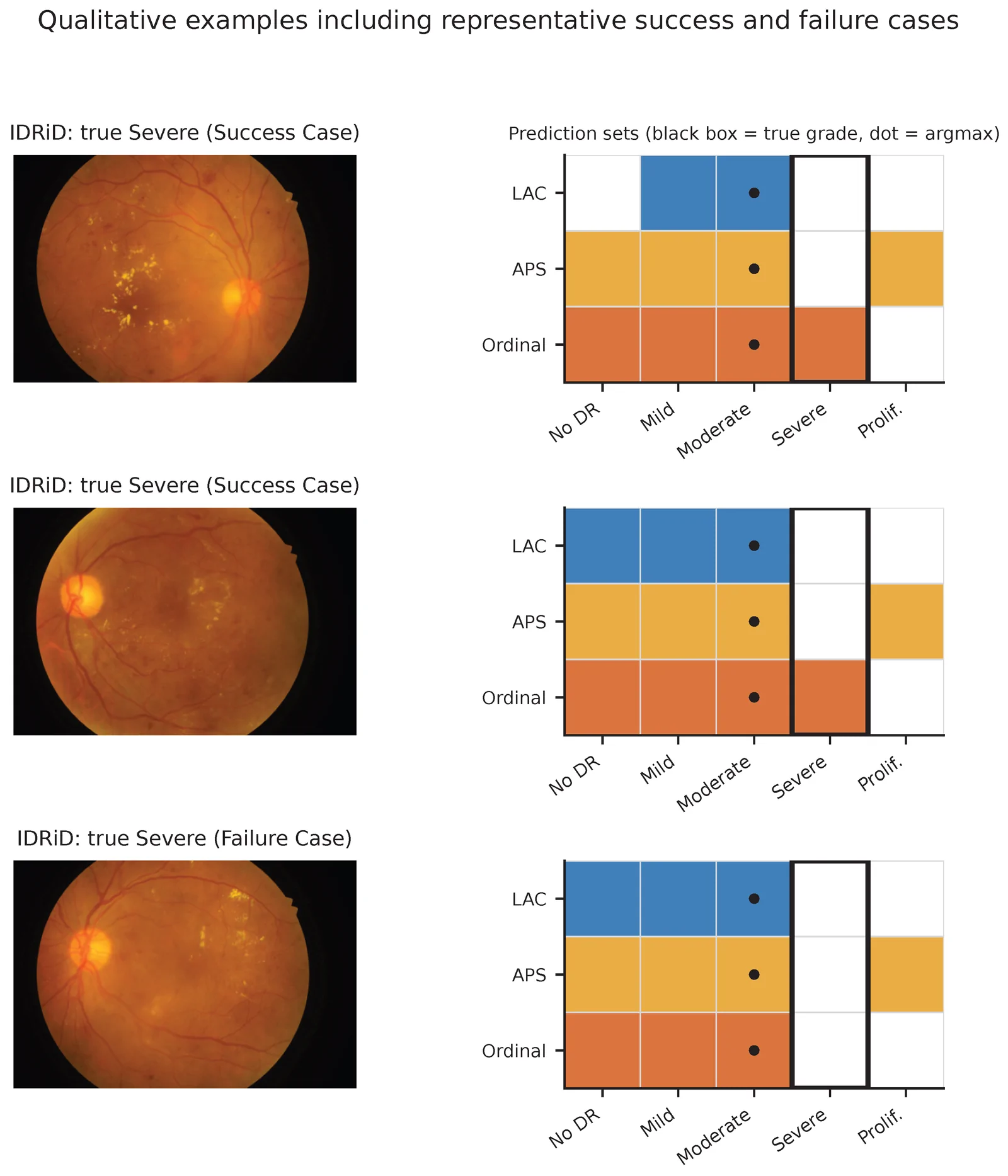
Representative IDRiD cases with true grade 3 (severe DR), including two ordinal successes and one ordinal failure. Each panel shows the prediction sets across the five grades (black outline: true grade; dot: point prediction). The failure case illustrates that contiguity does not guarantee inclusion of the true grade under dataset shift.

The contiguity constraint yields a concrete robustness benefit on the severe grade (Table 4; Figure 6 and Figure 7). Because a contiguous interval anchored near the prediction almost always brackets an interior grade (illustrated for individual IDRiD cases in Figure 8), the ordinal score achieves the highest grade-3 OOD coverage in every backbone–α setting. For EfficientNet-B0 at α=0.10, it reaches 0.710 [0.569,0.838] versus 0.431 [0.329,0.540] for the best baseline (APS), more than halving the coverage deficit relative to the 0.90 target (0.190 versus 0.469). The improvement is supported by the 95% bootstrap intervals: the ordinal interval does not overlap the best baseline’s at α=0.10 on either backbone, nor at α=0.05 on ResNet-50; at α=0.05 on EfficientNet-B0, the intervals are near-non-overlapping (ordinal lower bound 0.729 versus APS upper bound 0.771). Consistency is also seen per seed: the ordinal score attains the highest grade-3 OOD coverage in all five seeds in each setting.

The mitigation is substantial but not a full restoration of the guarantee. On the stronger ResNet-50 at α=0.05, the ordinal score brings grade-3 OOD coverage to 0.945 [0.883,0.990], approaching the 0.95 target, whereas on EfficientNet-B0 at α=0.10, it remains at 0.710, well below 0.90. Consistent with the interior-bracketing mechanism, the benefit does not extend to the boundary grade 4: there, the ordinal score is neutral-to-slightly-below the adaptive baselines (e.g., 0.688 versus 0.706 for APS on EfficientNet-B0 OOD at α=0.10).

### 4.10. Consistency Across Classifier Architectures

**Figure 9:**
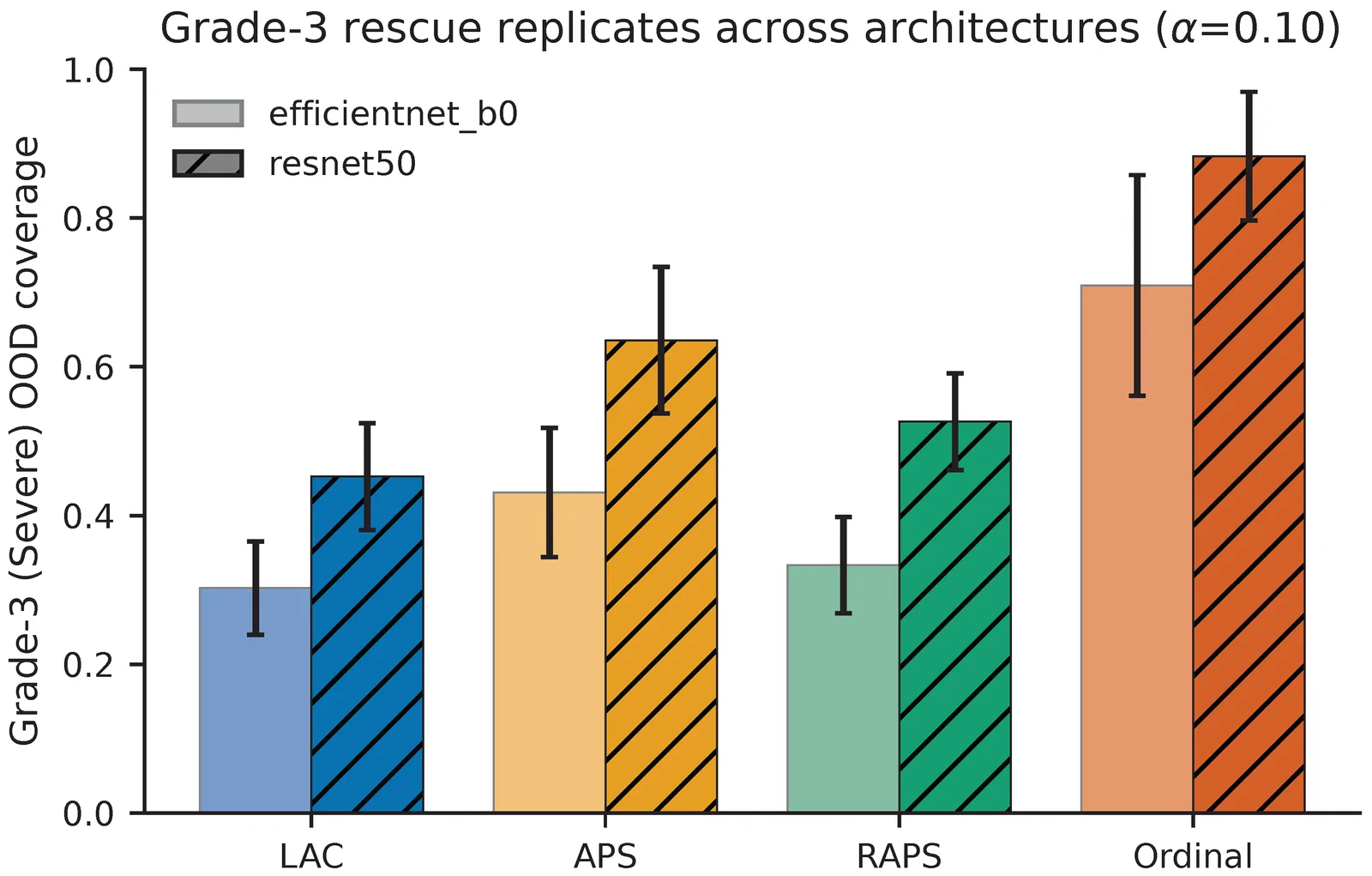
Grade-3 OOD coverage (α=0.10) for EfficientNet-B0 and ResNet-50, showing that the ordinal rescue replicates across architectures.

All four qualitative findings, approximately valid in-distribution coverage, external marginal degradation localized strongly to grade 3, exact ordinal contiguity, and improved grade-3 coverage, are replicated on EfficientNet-B0 and ResNet-50 (Figure 9). The independent ViT experiment in Table 4 shows the same direction of effect, providing evidence across convolutional and transformer backbones without establishing architecture-independent performance in general.

### 4.11. External Validation on IDRiD and Messidor-2

The external coverage degradation observed on IDRiD was also present in the independent ViT replication on Messidor-2. At the 90% target, APS coverage decreased from 0.920±0.023 internally to 0.632±0.134 on Messidor-2, while ordinal coverage was 0.676±0.178. At the 95% target, APS and ordinal coverage were 0.714±0.138 and 0.741±0.159, respectively. The ordinal method therefore improved marginal coverage modestly in this replication but remained well below both nominal targets. Messidor-2 contributes 75 additional severe-grade images; considered together, the two external cohorts contain 159 grade-3 images, although results were analyzed separately rather than pooled.

## 5. Discussion

This study evaluated whether calibrated prediction sets for AI-based DR grading remain reliable during external evaluation. Across two convolutional backbones and an independent ViT replication, internal coverage did not transfer uniformly to IDRiD or Messidor-2. On IDRiD, the largest class-specific deficit occurred in severe DR. Enforcing an ordinal interval produced structurally coherent outputs and improved severe-grade coverage, although it increased the average set size and did not restore a formal guarantee after dataset shift.

The results reinforce the importance of external validation in ophthalmic AI. Cross-camera studies have shown that DR software must be assessed across acquisition devices [10], while uncertainty-aware systems such as DR|GRADUATE have demonstrated that model uncertainty can provide information beyond a point grade [19]. Our findings extend this perspective by evaluating coverage-controlled prediction sets and showing that uncertainty reliability itself can fail across datasets. APTOS and IDRiD differ in image acquisition and case mix, including an increase in severe DR prevalence from approximately 5% to 18%. Consequently, an apparently acceptable external marginal coverage value may coexist with clinically important undercoverage in a specific disease stage.

The ordinal method changes how diagnostic ambiguity is communicated rather than retraining the image classifier. A contiguous set such as grades 2–3 indicates uncertainty between adjacent stages, while a disconnected set can be difficult to interpret. This constraint substantially reduced severe-grade undercoverage: with ResNet-50 at α=0.05, grade-3 coverage increased from 0.750 with APS to 0.945. The improvement was incomplete for EfficientNet-B0 at α=0.10, where grade-3 coverage increased from approximately 0.43 to 0.710 but remained below the 0.90 target. Ordinal structure should therefore be viewed as a diagnostic-output design choice that mitigates some failures, not as a correction for dataset shift.

The main strengths are the strict separation of development, calibration, and external evaluation data; replication across convolutional and transformer backbones; evaluation on two external cohorts; explicit grade-specific analysis; and bootstrap intervals incorporating image- and seed-level variability. The conformal scores were compared using identical base probabilities, calibration observations, and target coverage levels within each experiment.

The interval restriction and adjacent probability accumulation are jointly defined by the ordinal score rather than implemented as separately parameterized modules. Consequently, disabling the interval restriction changes the score itself instead of removing an independent post-processing component. APS serves as the operational unconstrained comparator, while future work could investigate alternative ordinal accumulation rules within the interval-valued family.

From a potential workflow perspective, a contiguous set can support a transparent upper-bound referral rule. For example, an output of {2,3} identifies adjacent moderate-to-severe ambiguity, whereas {1,3} does not define a coherent severity interval. A future clinical protocol could refer an image when the upper endpoint reaches a prespecified disease threshold. This interpretation is illustrative and requires prospective evaluation before clinical use.

Several limitations should be considered. This retrospective computational study used public benchmark datasets and two external evaluation cohorts. IDRiD and Messidor-2 contained 84 and 75 grade-3 images, respectively; the cohorts broaden the evidence but neither provides a large severe-grade sample. Patient-level demographic variables and standardized image-quality annotations were unavailable, limiting subgroup analysis. The study did not perform a valid same-model robustness experiment under blur, illumination, or contrast degradation, and it did not compare prediction sets with clinicians or prospective workflow outcomes. These limitations constrain clinical generalizability and motivate additional multicenter evaluation.

APTOS and IDRiD both originate from South Asian clinical settings. Messidor-2 adds geographic variation, but broader validation across populations, camera systems, and screening pathways remains necessary.

Future studies should evaluate ordinal conformal prediction prospectively across multiple clinical sites and camera systems, incorporate image-quality and patient-subgroup analyses, and compare prediction sets with clinician confidence or adjudicated disagreement. Methodological extensions could combine ordinal sets with shift-aware calibration, label-prior adjustment, or domain adaptation. Such evaluations would clarify the method’s potential role in referral, review, and diagnostic-support workflows.

## 6. Conclusions

Conformal prediction achieved approximately nominal internal coverage but became undercovered during evaluation on IDRiD and Messidor-2. On IDRiD, the failure concentrated in severe DR, demonstrating that aggregate uncertainty metrics can mask class-specific weaknesses.

Restricting prediction sets to adjacent DR grades produced fully contiguous outputs and improved severe-grade coverage across the tested convolutional and transformer experiments, with an increase in the number of returned grades. This provides a clearer representation of diagnostic ambiguity but does not eliminate the need for external calibration and validation.

For AI-assisted DR grading, uncertainty methods should be evaluated by disease stage and across datasets rather than through internal marginal performance alone. Prospective multicenter studies are required to determine whether ordinal prediction sets can support safer referral, review, or escalation workflows.

## Figures and Tables

**Table 1:** Summary of related work and positioning of the present study.

Research Area	Representative Focus	Limitation for This Problem	Role in This Study
Deep DR grading	Accurate fundus-image classifiers using CNNs, transformers, and lesion-aware representations	Often reports point predictions without calibrated set-valued uncertainty under deployment shift	Supplies the EfficientNet-B0, ResNet-50, and ViT-Base backbones for conformal calibration
Standard conformal prediction	Distribution-free marginal coverage using scores such as LAC, APS, and RAPS	Requires exchangeability and may produce non-contiguous sets on ordinal labels	Provides categorical baselines and the finite-sample calibration framework
Conformal prediction under shift	Covariate-shift reweighting and transductive or graph-based adaptation	Usually assumes stable conditional label mechanisms or additional unlabeled test data	Motivates evaluating coverage collapse under APTOS→IDRiD shift
Ordinal conformal prediction	Interval-valued prediction sets for ordered labels	Rarely evaluated for medical-image grading under combined covariate and label-prior shift	Provides the structural constraint used to improve contiguity and severe-grade robustness

**Table 2:** Marginal coverage, average set size, and contiguity fraction (mean ± std over five seeds) on the APTOS test split (ID) and IDRiD (OOD). Target coverage is 1−α.

			α=0.10			α=0.05		
Backbone	Dom.	Method	Cov.	Size	Contig.	Cov.	Size	Contig.
Eff.-B0	ID	LAC	0.905	1.197	0.955	0.949	1.519	0.903
		APS	0.928	1.364	0.908	0.962	1.679	0.895
		RAPS	0.917	1.268	0.921	0.955	1.591	0.884
		Ordinal	0.940	1.510	1.000	0.965	1.797	1.000
	OOD	LAC	0.657	1.386	0.913	0.789	1.932	0.860
		APS	0.710	1.646	0.863	0.827	2.170	0.840
		RAPS	0.669	1.476	0.869	0.803	2.021	0.830
		Ordinal	0.766	1.874	1.000	0.855	2.315	1.000
ResNet-50	ID	LAC	0.917	1.328	0.967	0.964	1.697	0.932
		APS	0.954	1.663	0.909	0.978	2.027	0.870
		RAPS	0.929	1.447	0.942	0.973	2.062	0.870
		Ordinal	0.958	1.767	1.000	0.974	2.102	1.000
	OOD	LAC	0.684	1.661	0.958	0.869	2.438	0.907
		APS	0.814	2.339	0.856	0.907	2.890	0.781
		RAPS	0.723	1.889	0.909	0.887	2.706	0.796
		Ordinal	0.858	2.472	1.000	0.939	3.031	1.000

Standard deviations are reported in the text; they are omitted here for legibility.

**Table 3:** Softmax calibration metrics for the independent five-seed ViT replication. Values are means over seeds and are shared across conformal methods.

Evaluation Cohort	ECE	NLL
APTOS test	0.150	0.936
IDRiD	0.076	1.688
Messidor-2	0.137	1.955

**Table 4:** Grade-3 (severe DR) coverage on IDRiD with 95% two-way cluster bootstrap confidence intervals (n=84). Values are means over five seeds; the target is 1−α. For the independent ViT replication, * and ** denote exact one-sided Wilcoxon evidence at p<0.10 and p<0.05, respectively.

Backbone	Method	α=0.10	α=0.05	% Improvement
Eff.-B0	LAC	0.302 [0.210, 0.402]	0.560 [0.450, 0.662]	–
	APS	0.431 [0.329, 0.540]	0.636 [0.490, 0.771]	–
	RAPS	0.333 [0.238, 0.429]	0.574 [0.462, 0.679]	–
	Ordinal	**0.710 [0.569, 0.838]**	**0.860 [0.729, 0.962]**	+64.7%/+35.2%
ResNet-50	LAC	0.452 [0.350, 0.560]	0.676 [0.583, 0.767]	–
	APS	0.636 [0.519, 0.748]	0.750 [0.643, 0.843]	–
	RAPS	0.526 [0.424, 0.629]	0.686 [0.564, 0.810]	–
	Ordinal	**0.883 [0.793, 0.960]**	**0.945 [0.883, 0.990]**	+38.8%/+26.0%
ViT-Base	LAC	0.344 [0.238, 0.452]	0.657 [0.548, 0.762]	–
	APS	0.416 [0.321, 0.512]	0.690 [0.583, 0.798]	–
	RAPS	0.356 [0.250, 0.464]	0.681 [0.571, 0.786]	–
	Ordinal	**0.773 [0.667, 0.857]** **	**0.850 [0.762, 0.929]** *	+85.8%/+23.2%

Bold marks the highest grade-3 coverage in each column. The percentage improvement compares the Ordinal score to the strongest baseline (APS) at α=0.10 and α=0.05, respectively.

## Data Availability

All datasets analyzed in this study are publicly available. The APTOS 2019 Blindness Detection dataset, with the training, validation, and test partitions used here, is available at https://www.kaggle.com/datasets/mariaherrerot/aptos2019 (accessed on 15 August 2026); the IDRiD (Indian Diabetic Retinopathy Image Dataset) disease-grading data are available at https://www.kaggle.com/datasets/mariaherrerot/idrid-dataset (accessed on 15 August 2026); the Messidor-2 data are available at https://www.kaggle.com/datasets/mariaherrerot/messidor2preprocess (accessed on 15 August 2026). The replication code is publicly available at https://github.com/umarbhasan/conformal-diabetic-retinopathy (accessed on 15 August 2026).
